# Periorbital changes associated with prostaglandin analogs in Korean patients

**DOI:** 10.1186/s12886-017-0521-4

**Published:** 2017-07-17

**Authors:** Hee Weon Kim, Youn Joo Choi, Kyung Wha Lee, Min Joung Lee

**Affiliations:** 1 Department of Ophthalmology, Hallym University College of Medicine, Hallym University Sacred Heart Hospital, 896 Pyeongchon-dong, Dongan-gu, Anyang, Gyeonggi-do 431-070 South Korea; 2 Department of Ophthalmology, Hallym University College of Medicine, Hallym University Kangdong Sacred Heart Hospital, Seoul, South Korea

**Keywords:** PAP, Periorbital change, PGA, Prostaglandin analog, Sulcus deepening

## Abstract

**Background:**

Prostaglandin analogs (PGAs) are commonly used to treat glaucoma because of their powerful intraocular pressure lowering effect. However, various periorbital changes associated with the use of PGAs have been reported. We investigated the incidence of periorbital changes in Korean patients who were treated with PGAs, and analyzed clinical factors associated with superior sulcus deepening.

**Methods:**

This study included 58 glaucoma patients who were treated with latanoprost, travoprost, or bimatoprost unilaterally. Face photographs were collected, and periorbital changes such as superior sulcus deepening, eyelid pigmentation, ptosis, lid retraction, dermatochalasis, and redness were evaluated by two oculoplastic specialists. For each patient, the contralateral eye served as a control. The frequency of ptosis, dermatochalasis, pigmentation, erythema, and superior sulcus deepening were analyzed. Demographic and ocular factors were compared between patients who showed superior sulcus deepening and those who did not.

**Results:**

Thirty-one patients (53.4%) showed one or more periorbital changes associated with PGAs. The most common change was superior sulcus deepening (24.1%), followed by eyelid pigmentation (19.0%), eyelid erythema (19.0%), dermatochalasis (10.3%), eyelid retraction (5.2%), and ptosis (3.4%). The age of the patient and the duration of PGA administration was significantly correlated with superior sulcus deepening (*p* = 0.007, *p* = 0.002, respectively).

**Conclusions:**

Periorbital changes are frequently seen in patients who use PGAs, and superior sulcus deepening is the most common change in Korean patients. Long-term use of PGAs and old age were associated with superior sulcus deepening.

## Background

Prostaglandin analogs (PGAs) are popular first-line drugs for glaucoma treatment for many reasons: they powerfully reduce intraocular pressure, are associated with few systemic adverse reactions, and need to be applied only once a day. However, they may cause periorbital changes, which result in cosmetic problems and are considered as a main disadvantage of using these drugs. Collectively known as prostaglandin-associated periorbitopathy (PAP), these changes include superior sulcus deepening, orbital fat atrophy, ptosis, and dermatochalasis [1]. The cause or mechanism underlying PAP is not entirely clear, but thought to be related to the effect of PGF2a on adipocytes. Suppression of adipogenesis, inhibition of preadipocyte proliferation and adipocyte differentiation were suggested as possible pathophysiologic mechanisms in in vitro studies [2–4]. In addition, other periocular soft tissue changes such as eyelid pigmentation, eyelid erythema, eyelash growth, and blepharitis can occur with the use of PGAs [5].

In 2004, Peplinski and Albani Smith [6] first reported three patients who showed alteration of eyelid appearance with deepening of the eyelid sulcus after topical bimatoptost therapy, and this adverse event was also reported sequentially in travoprost, latanoprost, and tafloprost [7–9]. To date, there have been several studies about PAP that focus primarily on the incidence of the condition, and the results are inconsistent [9–14]. The differences in study design and inclusion criteria of the subjects, the racial differences among the subjects, and the subjective judgment of periorbital changes are possible reasons for the variations in the results. In addition, the clinical risk factors predicting PAP development were rarely investigated.

The purpose of this study was to investigate the frequency of common PAP in Korean patients who were treated with PGAs in one eye, and analyze the relationship between superior sulcus deepening and various clinical factors.

## Methods

We performed a retrospective medical record review of patients who had been treated with latanoprost, travoprost, or bimatoprost at the Hallym University Sacred Heart Hospital between April 2014 and December 2014. Fifty-eight patients (39 men and 19 women) who were diagnosed with glaucoma were included. The PGA eyedrop was administered in only 1 eye. All the subjects were treated with 1 of the 3 PGA eyedrops for ≥3 months. None of the patients had used other PGAs before the assessment. Subjects were excluded if they had any previous ophthalmic surgeries other than phacoemulsification and intraocular lens implantation, or if they had previous trauma or surgical history of ocular adnexa. Subjects who had thyroid orbitopathy or used contact lenses were also excluded. Written informed consent was obtained from each participant prior to enrollment in this cross-sectional retrospective study.

For each enrolled patient, a photograph of the upper face was taken without a flash by using a single-lens digital camera (EOS 500D, Canon, USA). All photographs were taken under the same room conditions, and the contralateral eye of each patient served as a control. The presence of the periorbital changes was evaluated by 2 oculoplastic specialists. They compared the treated eye with the untreated one in a picture of each patient, and to determine the presence of superior sulcus deepening, eyelid pigmentation, ptosis, lid retraction, dermatochalasis, and redness. Superior sulcus deepening was evaluated on a scale of 0 to 4 as follows: grade 0 = none; grade 1 = trace, barely visible; grade 2 = mild; grade 3 = moderate, easily detected; grade 4 = severe. A grade of 2 or more was considered significant sulcus deepening [15]. Ptosis and upper eyelid retraction were defined as a 2 mm or more height difference between the eyelids. If there was a disagreement, the final judgment was decided by mutual agreement. Hertel exophthalmometer measurements were obtained, and a difference of 2 mm or more between the two eyes was considered significant finding.

Electronic medical records were reviewed, and the following demographic and clinical data were collected: age, gender, systemic diseases, type of glaucoma, history of cataract surgery, type of PGA, duration of PGA administration, baseline intraocular pressure (IOP), effect of PGA on IOP reduction, combination with other glaucoma topical eyedrops, and severity of glaucoma. The type of glaucoma was classified as normal-tension glaucoma (NTG), primary open-angle glaucoma (POAG), or other. To evaluate the effect of PGAs on lowering IOP, the base IOP was compared with IOP at 3 months after PGA use. The severity of glaucoma was judged quantitatively by the visual field index (VFI).

The frequency of each item was described, and the risk factors for superior sulcus deepening were analyzed. The Mann-Whitney U test and Fisher’s exact test were used to compare patients with superior sulcus deepening to those without the condition. Univariate and multivariate logistic regression were performed to estimate the associations between different variables and superior sulcus deepening. A probability value of <0.05 was considered statistically significant. All the analyses were performed using SPSS version 22.0 for Windows (SPSS Inc., Chicago, IL, USA).

This study was performed in accordance with the Declaration of Helsinki and was approved by the Institutional Review Board of the Hallym University Sacred Heart Hospital (IRB No: 715167).

## Results

A total of 58 patients were included in this study. The mean age of patients was 61.05 ± 5.81 years (range, 15–86), and the mean duration of treatment was 26.76 ± 31.25 months (range, 3–133). In total, 50 patients were included in the latanoprost group, 4 patients in the bimatoprost group, and 4 patients in the travoprost group. NTG was the most common type of glaucoma (65.5%). Twenty-three patients (39.7%) were treated with combination of other eyedrops. The detailed patient characteristics are shown in Table 1.

**Table 1:** General characteristics of patients

Numbers of patients (eyes)	58
Age (years)	61.05 ± 15.81
Gender (male: female)	39: 19
Duration of treatment (months)	26.76 ± 31.25
Pre-treatment IOP (mmHg)	19.17 ± 6.70
IOP lowering effect (%)	25.03 ± 18.47
Visual field index	81.22 ± 22.91
History of cataract surgery (%)	17 (29.31)
Type of glaucoma (%)	
NTG: POAG: Others	38(65.51): 9(15.52): 11(18.97)
Type of Prostaglandin analogs	
Latanoprost: Bimatoprost: Travoprost	50(86.21): 4(6.90): 4(6.90)
Combination of other eyedrops (%)	23 (39.66)

IOP = Intraocular pressure; NTG = Normal tension glaucoma; POAG = Primary open angle glaucoma

**Fig. 1:**
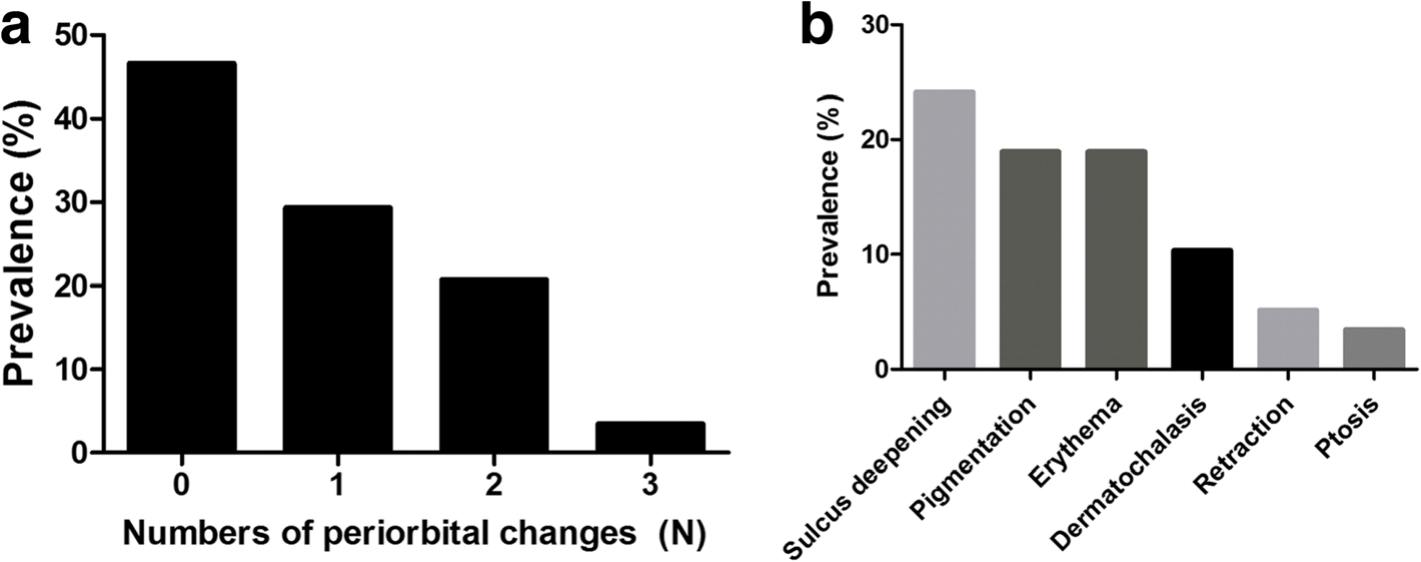
Prevalence of periorbital changes. **a** 31 patients (53.4%) showed one or more periorbital changes associated with PGA. **b** Of the periorbital changes, superior sulcus deepening was observed most commonly

**Fig. 2:**
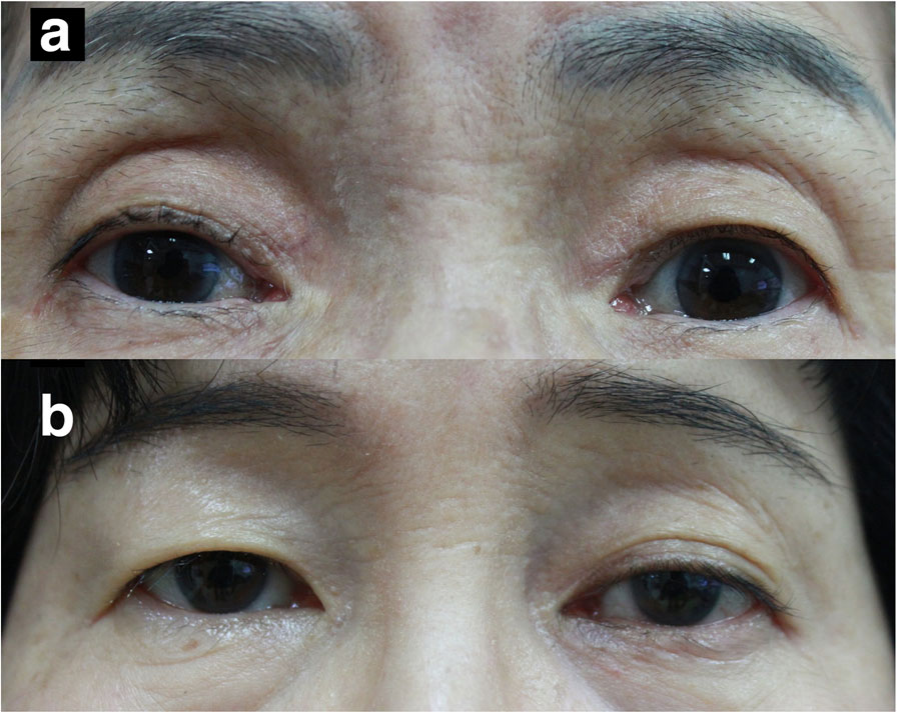
Representative cases. **a** Facial photograph of 75 year-old female patient 6 months after treatment of right eye with topical latanoprost. Compared to the left side, the right upper eyelid shows deeper sulcus and greater pigmentation. **b** Eyelid photograph of 60 year-old female patient 17 months after treatment of left eye with topical bimatoprost. Superior sulcus deepening and significant ptosis is observed on her left upper eyelid. Note larger eyelid crease and brow elevation on the left side

Thirty-one patients (53.4%) showed 1 or more periorbital changes associated with PGAs, 12 patients (20.7%) showed 2 periorbital changes, and 2 patients showed 3 periorbital changes (Fig. 1a). The most common periorbital change was superior sulcus deepening (24.1%), followed by eyelid pigmentation (19.0%), eyelid erythema (19.0%), dermatochalasis (10.3%), upper eyelid retraction (5.2%), and ptosis (3.4%) (Fig. 1b). These positive findings could be easily judged by comparing the affected eye with the contralateral eye in face photograph (Fig. 2). In addition, asymmetric enophthalmos ≥2 mm was noticed in 5 of 42 patients (11.9%).

Table 2 presents the correlation between the demographic and clinical factors and superior sulcus deepening. Patient age and the duration of PGA administration showed significant difference between patients with or without superior sulcus deepening (*p* = 0.007 and *p* = 0.002, respectively, Mann-Whitney U test). History of cataract surgery and the severity of glaucoma judged by VFI showed borderline significance (*p* = 0.056 and *p* = 0.085, respectively, Fisher’s exact test and Mann Whitney U test). The results of the univariate and multivariate logistic regression analyses indicated that the age of the patient and the duration of PGA administration were independent risk factors for superior sulcus deepening (OR 1.083, *p* = 0.016 and OR 1.034, *p* = 0.010, respectively, Table 3).

**Table 2:** Correlation between demographic and clinical factors and superior sulcus deepening

	Superior sulcus deepening		* p*-value
	Yes (*n* = 14)	No (*n* = 44)	
Age (years)	69.57 ± 8.97	56.95 ± 16.88	0.007*
Gender (male: female)	7:7	32:12	0.189†
Duration of administration (months)	49.50 ± 39.61	19.52 ± 24.46	0.002*
Pre-treatment IOP (mmHg)	17.57 ± 3.52	19.68 ± 7.39	0.512*
IOP lowering effect (%)	18.79 ± 16.14	27.98 ± 17.16	0.134*
Visual field index	73.86 ± 25.64	83.57 ± 21.76	0.085*
History of cataract surgery (%)	50.00	22.73	0.056†
Type of glaucoma (%)			
NTG: POAG: Others	9(64):4(29):1(7)	29(66):5(11):10(23)	0.228†
Type of prostaglandin analogs (%)			
Latanoprost: Bimatoprost: Travoprost	12(86):1(7):1(7)	38(86):3(7):3(7)	0.999†
Combination of other eyedrops (%)	42.86	38.64	0.508†

IOP = Intraocular pressure

* Mann-Whitney U test

† Fisher exact probability test

**Table 3:** Multivariate analysis for superior sulcus deepening

	Odd ratio (95% CI)	*p*-value
Univariate analysis		
Age (years)	1.076 (1.013–1.142)	0.017
Duration of administration (months)	1.029 (1.008–1.051)	0.007
History of cataract surgery (%)	3.400 (0.962–12.020)	0.058
IOP lowering effect (%)	0.963 (0.923–1.006)	0.088
Multivariate analysis		
Age (years)	1.083 (1.015–1.156)	0.016
Duration of administration (months)	1.034 (1.008–1.060)	0.010
History of cataract surgery (%)	-	0.781
IOP lowering effect (%)	-	0.124

IOP = Intraocular pressure; CI = confidence interval

## Discussion

In this study, 53.4% of the subjects (31 of 58) showed one or more sign of PAP after a mean of 27 months of using PGAs. The most common sign of PAP was superior sulcus deepening, followed by eyelid pigmentation, eyelid erythema, dermatochalasis, upper eyelid retraction, and ptosis. In many studies, superior sulcus deepening has been reported as a common change, although the frequency was variable. Custer et al. [13] reported that superior sulcus deepening developed in 68.5% patients who were using PGAs. Kucukevcilioglu et al. [12] reported superior sulcus deepening in 80%of patients using bimatoprost, 45% of patients using travoprost, and 15.7% of patients using latanoprost. Inoue et al. [10] also analyzed the frequency of superior sulcus deepening with respect to the type of PGA, and they reported that it was observed in 24% of the latanoprost group, 50% of the travoprost group, 18% of the tafloprost group, and 60% of the bimatoprost group. Generally, bimatoprost is known to show higher incidence of superior sulcus deepening than latanoprost or travoprost [10, 16, 17]. However, in this study, no statistically significant differences were seen between sulcus deepening and the types of PGA, because most of the subjects used latanoprost (86%, 50 of 58 subjects). Although latanoprost is first-released and most widely used PGA, there are a few studies that reported deep superior sulcus after the use of latanoprost [10, 11, 14]. In addition, Asian patients can be more sensitive to the deepening of superior sulcus, because Asians usually do not have superior sulcus depression. The prevalence of superior sulcus deepening in this study was higher (24% with 27 months of use) than in another latanoprost-dominant Japanese study; Nakakura et al. [18] investigated the incidence of PAP in patients treated with latanoprost and reported that superior sulcus deepening was observed in 13.6% of patients within a 26 month follow-up period (Table 4). In the current study, superior sulcus deepening was assessed by oculoplastic specialists, and we adopted a grading system for evaluation. The methodology differences between the two studies may explain the differences in the results.

**Table 4:** Comparison of the prevalence of superior sulcus deepening between previous latanoprost-dominant Asian studies and this study

	Inoue et al. [10]	Yoshino et al. [11]	Nakakura et al. [18]	This study
Number of eyes	50	39	22	58
Laterality	Unilateral	Unilateral	Unilateral or bilateral	Unilateral
Type of PGA	L	L:TV:TF:B = 23:9:6:1	L	L:TV:B = 50:4:4
Age (years)	62.1 ± 12.3	65.5 ± 10.2	60.3 ± 12.2	61.05 ± 15.81
Duration of administration (months)	60.0 ± 32.4	67.7 ± 46.7	26.0 ± 8.2	26.76 ± 31.25
Superior sulcus deepening (%)	24%	53.9%	13.6%	24.1%

PG = prostaglandin analog; L = latanoprost; TV = travoprost; TF = tafloprost; B = bimatoprost

We tried to find the relationship between superior sulcus deepening and demographic and clinical factors. We found that age and duration of PGA use were positively and independently associated with development of superior sulcus deepening. The authors of some studies implicitly commented that the duration of PGA administration may affect the frequency of superior sulcus deepening, but few studies have indicated that the duration of PGA use is an independent risk factor for superior sulcus deepening, although it is a reasonable assumption. Yoshino et al. [11] previously reported that superior sulcus deepening was positively correlated with the duration of PGA use. Similarly, in this study, patients with superior sulcus deepening had used PGA significantly longer than those without superior sulcus deepening (*p* = 0.002, Mann-Whitney U test), and the odds of having superior sulcus deepening were 3.4% higher for each year of PGA administration.

In the current study, in addition to the duration of PGA use, the age of the patient was significantly associated with the deepening of the superior sulcus. Age was also suggested as a risk factor for superior sulcus deepening in a previous Japanese study [16]. The researchers of that study investigated the factors related to the occurrence of superior sulcus deepening associated with bimatoprost, and they reported the incidence was significantly higher in older patients and in patients who had nonmyopic eyes. Superior sulcus deepening after PGA administration has been attributed to orbital fat atrophy and has been reported in histologic and imaging studies [19–22]. Depletion of intraorbital fat is also acharacteristic age-related change, along with redistribution of orbital fat and descent of suspensory ligaments [23]. Use of PGA may aggravate orbital fat atrophy in addition to the aging change.

Ptosis was found in 3.4% of the patients in this study, whereas upper eyelid retraction was found in 5.2% of the patients. Ptosis has been described as a common sign of PAP [12, 18]. However, there are some controversies with regard to this interpretation. Ptosis can develop in the elderly because of involutional changes; hence, the frequency of ptosis can be easily overestimated, especially when analyzing bilaterally treated cases. For this reason, some researchers insist that ptosis is not necessarily a sign of PAP, but rather an independent age-related finding [24]. In addition, dermatochalasis can be mistaken as ptosis, especially in photographs. In one study, researchers commented that ptosis displayed poor agreement among 3 observers [18]. We analyzed unilaterally treated cases, and gave attention to differentiate dermatochalasis and ptosis.

Upper eyelid retraction was recently reported in a few studies. Noma and Kakizaki [16, 25] reported a case in which bilateral upper eyelid retraction was associated with the use of bimatoprost. Recently, Rabinowitz et al. [24] investigated the signs of PAP in patients treated with PGA unilaterally, and they reported that upper eyelid retraction was found in 76% of patients (25 out of 33). They compared the marginal reflex distance 1 (MRD1) between the treated and the untreated eyes, and the average amount of retraction was 1.49 mm. We defined the upper eyelid retraction as the MRD1 of the treated eye being at least 2 mm higher than the MRD1 of the untreated eye considering normal variation and asymmetry before PGA use. Such a strict criterion can explain the lower prevalence of upper eyelid retraction in this study. The mechanism underlying upper eyelid retraction is still unknown, although inflammation, dorsal traction by sunken superior sulcus, and fibrosis of the levator muscle have been suggested. In this study, 1 of 3 patients showing upper eyelid retraction simultaneously showed superior sulcus deepening.

Eyelid pigmentation was observed in 19% of patients (11 of 58). The prevalence of eyelid pigmentation was reported to be between 0 and 61.5% [5, 11, 13, 18, 26]. The racial difference and the duration of PGA administration may explain the differences in the results. Eyelid pigmentation occurred more frequently in Asians than in Caucasians. Custer et al. [13] retrospectively reviewed PAP in 35 patients using PGAs, and they reported that eyelid pigmentation was not grossly recognized in their case series. In a Swedish study analyzing the safety of a latanoprost/timolol fixed combination, eyelid pigmentation was reported in 6.2% of patients at 60 months of administration [26]. However, Yoshino et al. [11] reported that eyelid pigmentation was noticed in 61.5% of Japanese patients who used PGAs for a mean of 67.7 months. They suggested that the melanocytes of Asians could be more susceptible to PGAs. The duration of PGA administration may have influenced the frequency of eyelid pigmentation. We included subjects who were treated with PGAs for more than 3 months, and the mean duration was 27 months. The shorter duration may explain the lower prevalence of eyelid pigmentation (19%) in our study than in the previous Japanese study [11]. However, Inoue et al. [5] compared patients with and without eyelid pigmentation, and they reported that there was no difference between the two groups with respect to the administration periods of PGA.

This study has some strengths and weaknesses. To prove the effect of PGAs on periorbital changes, it would be ideal to compare photos before and after the treatment. However, the pretreatment clinical data or photos were not available due to the retrospective nature of this study. Instead, we enrolled monocular PGA users, and strictly excluded patients who had a history of periorbital trauma or surgery, patients with thyroid orbitopathy and contact lens users. All of the patients were being treated unilaterally, and this is very important point to differentiate true PAP from individual variation or simple aging change, as commented in other studies [12, 24]. We believe that the patient’s untreated eye can sufficiently serve as a control. We tried to measure periorbital changes quantitatively, although the evaluation of PAP is inevitably subjective. We adopted a grading system for superior sulcus deepening, and set a strict point to define ptosis and upper eyelid retraction. However, with regard to erythema and pigmentation, we could not set a standard value because of differences among patients in skin tone and texture. However, all cases of PAP were evaluated by oculoplastic specialists who were familiar with periorbital changes but had not been given any clinical information. Our study was limited because most of the patients used latanoprost. Other limitations in our study was the short follow-up period.

## Conclusions

In conclusion, we demonstrated that several signs of PAP, including superior sulcus deepening, eyelid pigmentation, erythema, retraction, and ptosis, can occur in Korean patients. Although PGAs are convenient, tolerable, and effective topical medications for glaucoma, clinicians should keep these periorbital changes in mind whenever they prescribe PGAs. Long duration of PGA use and old age are significantly related to the development of superior sulcus deepening, which is the most common sign of PAP. Therefore, regular oculoplastic examination with sequential photographing will be helpful to detect the side effect earlier, especially in elderly patients.

## Abbreviations

CI: Confidence interval
IOP: Intraocular pressure
MRD1: Marginal reflex distance 1
NTG: Normal-tension glaucoma
PAP: Prostaglandin-associated periorbitopathy
PGAs: Prostaglandin analogs
POAG: Primary open-angle glaucoma
VFI: Visual field index
